# Improvement in Eating Disorder Risk and Psychological Health in People with Class 3 Obesity: Effects of a Multidisciplinary Weight Management Program

**DOI:** 10.3390/nu13051425

**Published:** 2021-04-23

**Authors:** Milan K. Piya, Ritesh Chimoriya, William Yu, Kathy Grudzinskas, Kyaw Phone Myint, Kathryn Skelsey, Nic Kormas, Phillipa Hay

**Affiliations:** 1 School of Medicine, Western Sydney University, Campbelltown, NSW 2560, Australia; r.chimoriya@westernsydney.edu.au (R.C.); kyaw09@gmail.com (K.P.M.); p.hay@westernsydney.edu.au (P.H.); 2 South Western Sydney Metabolic Rehabilitation and Bariatric Program, Camden Hospital, Camden, NSW 2570, Australia; william.yu@health.nsw.gov.au (W.Y.); kathy.grudzinskas@health.nsw.gov.au (K.G.); kathryn.skelsey@health.nsw.gov.au (K.S.); nic.kormas@health.nsw.gov.au (N.K.); 3 Translational Health Research Institute, Western Sydney University, Campbelltown, NSW 2560, Australia

**Keywords:** eating disorders, psychological distress, health-related quality of life, class 3 obesity, weight management

## Abstract

This study aimed to evaluate the risk of eating disorders, psychological distress, and health-related quality of life (HRQoL) in people with class 3 obesity (body mass index (BMI) ≥ 40 kg/m^2^), and the effect of multidisciplinary weight management over 12 months. This retrospective cohort study included all adults with class 3 obesity who enrolled in a weight management program from March 2018 to December 2019. Questionnaires included the Eating Disorder Examination Questionnaire Short (EDE-QS), Kessler Psychological Distress Scale (K10), and 36-Item Short Form Survey (SF-36) for HRQoL. Physical and Mental Component Summary scores (PCS and MCS) were derived from the SF-36. Of 169 participants who completed 12 months in the program, 65.7% (*n* = 111) completed questionnaires at baseline and 12 months, with 6.0 ± 6.8% weight loss over this period. Compared to baseline, there was significant improvement at 12 months in EDE-QS (15.7 ± 6.6 vs. 13.6 ± 6.2, *p* = 0.002), K10 (25.7 ± 9.7 vs. 21.2 ± 9.4, *p* < 0.001), PCS (29.4 ± 10.1 vs. 36.1 ± 10.9, *p* < 0.001), and MCS scores (40.2 ± 12.4 vs. 44.0 ± 13.4, *p* = 0.001). All, apart from EDE-QS scores, remained significant after adjusting for weight change. This study highlights the importance of multidisciplinary management in people with class 3 obesity to help reduce eating disorder risk and psychological distress, and improve HRQoL, in addition to weight loss.

## 1. Introduction

The increasing global prevalence of class 3 obesity (body mass index (BMI) ≥ 40 kg/m^2^) is a major public health problem [1,2,3]. In Australia, overweight and obesity has affected approximately two-thirds of adults, while 4% have class 3 obesity [4]. An increasing BMI is strongly associated with the amplification of both health risks and healthcare expenditure [3,5]. The evidence for physical comorbidities to be associated with obesity, such as type 2 diabetes mellitus (T2DM), non-alcoholic fatty liver disease (NAFLD), cardiovascular disease (CVD), and obstructive sleep apnoea (OSA), is well established [6,7,8], and weight loss has been linked to improved outcomes in people with these physical comorbidities [9,10,11,12]. This has led to an increasing number of patients opting to undergo bariatric surgery to aid weight loss, with over 630,000 procedures performed annually worldwide [13], and over 20,000 per year in Australia alone [14]. There is also an increasing recognition of the link between obesity and mental health issues such as depression, anxiety, and eating disorders [15,16,17,18], including in a large number of people who present for bariatric surgery or attend weight management clinics [19,20]. There is also a strong inverse relationship between BMI and quality of life [21], with the presence of class 2 and 3 obesity (BMI ≥ 35 kg/m^2^) being linked to a significant reduction in quality of life, even in the absence of chronic diseases [22]. However, while the improvements in physical comorbidities following bariatric surgery have been demonstrated in the short- as well as long-term [23,24,25], the mental health outcomes, eating disorder status, and quality of life following bariatric surgery or weight loss are less clear [18].

The prevalence of eating disorders and their associated symptoms is increasing globally [15,26,27,28], creating the potential for major personal and public health impacts [29]. Characterised by abnormal eating behaviours and psychological disturbances, eating disorders have notable impacts on physical health [30]. Currently recognised eating disorders include anorexia nervosa (AN), bulimia nervosa (BN), binge eating disorder (BED), as well as atypical anorexia, which has recently been added to the DSM-5 list of diseases to describe anorexia nervosa in the absence of low BMI [30]. The occurrence of eating disorders and obesity is interlinked, and they often coexist in the same individual [18,31]. Existing evidence on the association between eating disorders and weight disorder indicates a bidirectional relationship of both risk and maintenance factors [32,33,34]. Obesity is both a risk factor and a common outcome for individuals with eating disorders, particularly BN and BED [18,28]. Among individuals seeking treatment for severe obesity, a high prevalence of BED (up to 50%) has been reported [35]. In Australia, 13.5% of bariatric surgery candidates were found to have BED, while subthreshold disordered eating behaviours in the presurgical population are likely to exceed these rates [18]. Since individuals with both obesity and eating disorders are at a higher risk of psychosocial and medical consequences [19,36], and people with obesity are more likely to seek bariatric surgery [37], an improved understanding of this complex inter-relationship is imperative [31].

A multidisciplinary model of care is highly recommended for patients with class 3 obesity [38]. A recent study in the same weight management program reported significant weight loss in patients with class 3 obesity at six months [39]. However, the role of multidisciplinary management and weight loss on the risk of eating disorders, psychological distress, and quality of life is not well understood in this population. Therefore, this study aimed to:

1. Evaluate the risk of eating disorders, level of psychological distress, and health-related quality of life (HRQoL) in people with class 3 obesity.
2. Explore the 12-month outcomes of eating disorder risk, psychological distress, and HRQoL in people with class 3 obesity attending a multidisciplinary weight management program.

## 2. Methods

### 2.1. Study Design

This was a retrospective cohort study conducted in a hospital-based, publicly funded, multidisciplinary weight management program in Sydney, Australia, as previously described [39,40]. In brief, all patients enrolled in the program were over 18 years of age with a BMI ≥ 40 kg/m^2^ and at least one weight-related medical comorbidity, most often T2DM or NAFLD. The multidisciplinary team consisted of endocrinologists, a gastroenterologist, dietitians, a psychiatrist, psychologists, physiotherapists, and a specialist nurse. The clinical team provided individualised care to each patient, which included one or a combination of the following interventions: lifestyle interventions, behavioural modifications, dietary interventions, and pharmacotherapy. Some patients in this cohort were keen on bariatric surgery, some were not, and some were undecided. None of the patients underwent bariatric surgery within this 12-month period and received similar care regardless of their preference for bariatric surgery. All patients were advised to see the psychologist, and those who were thought to be at high risk of eating disorders or mental health problems by the team were referred to the psychiatrist. All patients newly enrolled in the program between March 2018 and December 2019, who attended at least one physician appointment, were included in this study.

### 2.2. Data Collection and Measurement

Data on weight, medical comorbidities, medications, and blood tests were collected from routine clinic data at the initial presentation to the clinic at baseline, and after 12 months of follow-up, where available. All patients were asked to complete three questionnaires as a part of routine clinical care at these time points. All patients with complete data available for baseline and 12 months, also had the number of visits with a health professional in the program, as well as the number of visits with the psychologist, recorded. The number of patients who saw the psychiatrist in the program was also recorded. The questionnaires included: Eating Disorder Examination Questionnaire Short (EDE-QS) to assess risk of eating disorders; Kessler Psychological Distress Scale (K10) to assess psychological distress; and 36-Item Short Form Survey (SF-36) to assess HRQoL. The study was approved by the South West Sydney Local Health District (SWSLHD) Research Ethics Committee as a quality improvement project (Reference: CT22_2018). Each questionnaire is described below.

#### 2.2.1. Risk of Eating Disorders (EDE-QS)

EDE-QS was developed as a 12-item version of the Eating Disorder Examination Questionnaire (EDE-Q), for use in sessional outcome monitoring [36]. The Cronbach’s alpha for EDE-QS was 0.80, indicating good internal consistency. EDE-QS assesses the frequency or severity of core eating disorder symptoms for the main disorders, AN, BN, and BED, over the past seven days [41]. The responses to the 12 items are recorded on a four-point rating scale, ranging from 0 = “0 days” or “not at all” to 3 = “6–7 days” or “markedly”. The scores for each of the items are summed to obtain a total score of 0 to 36, where higher scores indicate more frequent and severe eating disorder symptoms, and thus a higher risk of eating disorder [36,41]. While screening for clinically significant eating disorder symptoms using the EDE-QS, a cut-off point of 15 has been proposed as a threshold score to identify a high risk of eating disorder [41].

#### 2.2.2. Psychological Distress (K10)

K10 is a scale of non-specific psychological distress [42], widely used in health surveys for epidemiological and clinical purposes [43]. The Cronbach’s alpha for K10 was 0.94, indicating excellent internal consistency. The 10-item questionnaire includes questions about negative mood, anxiety, and emotional states to quantify the severity and frequency of those symptoms over the previous four-week period [44]. The items are rated on a five-point Likert scale, ranging from 1 = “none of the time” to 5 = “all of the time”. The scores for each of the items are summed to obtain a total score of 10 to 50, where higher scores indicate higher levels of psychological distress [45]. The levels of psychological distress based on the total K10 score are: low (10–15), moderate (16–21), high (22–29), and very high (30–50) [45].

#### 2.2.3. Quality of Life (SF-36)

SF-36 is a frequently used tool to assess HRQoL [46]. The Cronbach’s alpha for SF-36 was 0.94, indicating excellent internal consistency. SF-36 consists of eight multi-item subscales measuring physical function (10 items), role limit physical (4 items), role limit emotional (3 items), vitality (4 items), mental health (5 items), social function (2 items), bodily pain (2 items), and general health (5 items) [47]. The eight subscales can be aggregated into two component summary scales: the Physical Component Summary (PCS) score to assess physical health and the Mental Component Summary (MCS) score to assess mental health [48]. The component summary scores range from 0 to 100, where higher scores indicate better quality of life.

In this study, using standard SF-36 scoring algorithms [47], the scores for all eight subscales were calculated based on the responses to the items on each scale. The scores for each subscale were then standardised using a z-score transformation [48] and SF-36 scale means and standard deviations for Australian adult population norms [49] accessed from the Australian Bureau of Statistics. Finally, the standardised scores of the eight subscales were aggregated to calculate the PCS and MCS scores.

### 2.3. Statistical Analysis

The Statistical Package for Social Sciences, Version 25 (SPSS for MacOS, SPSS Inc., Chicago, IL, USA), was used for statistical analysis. Baseline data were available for all participants, but only participants who completed 12-month follow-up were included in the data analysis comparing baseline with 12-month data. The Kolmogorov–Smirnov test was performed to examine the normal distribution of the data. Additionally, the Breusch–Pagan test was performed to ensure homoscedasticity. A paired sample *t*-test was conducted to determine if there was a significant difference between the mean scores at baseline and 12-month follow-up. Analysis of covariance (ANCOVA) was conducted to adjust the scores for weight and number of health professional contacts over 12 months. Additionally, Pearson’s correlation coefficient was calculated to measure the strength and direction of the linear relationship between all the scores. Results were reported as percentages and mean ± standard deviations.

## 3. Results

A total of 262 participants enrolled in the South Western Sydney Metabolic Rehabilitation and Bariatric Program (SWS MRBP) between March 2018 and December 2019 and were included in this study. Baseline characteristics of the study participants are presented in Table 1. The baseline BMI of study participants was 51.2 ± 9.0 kg/m^2^, and they had a high risk of eating disorders (EDE-QS score: 16.2 ± 6.7), high levels of psychological distress (K10 score: 26.0 ± 9.6), and a poor quality of life (PCS score: 30.8 ± 10.3; MCS score: 39.3 ± 11.8). The correlation of PCS and MCS score with baseline weight was not statistically significant for either score, whereas K10 score (*r* = 0.16, *p* = 0.012) and EDE-QS score (*r* = 0.12, *p* = 0.050) had a statistically significant correlation with baseline weight.

**Figure 1:**
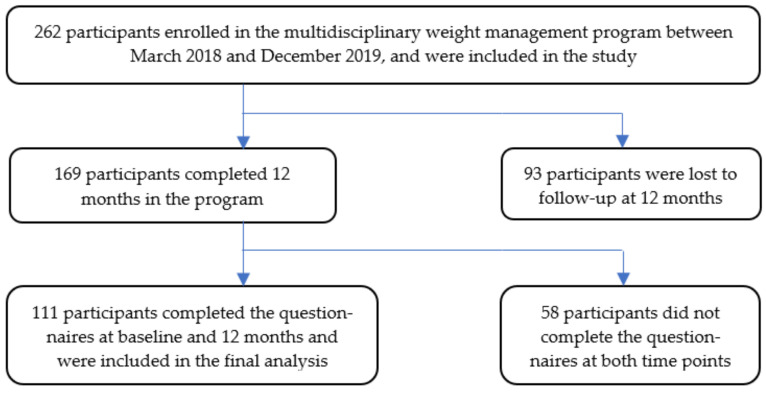
Entry of patients at baseline and loss to follow-up at 12 months.

Of the 262 participants, 64.5% (*n* = 169) had completed 12 months in the program. Of these, 65.7% (*n* = 111) had completed the questionnaires at baseline and 12 months (Figure 1). Compared to those who did not have complete data at 12 months (*n* = 151), those who had follow-up visit data and questionnaires completed at 12 months (*n* = 111) had similar baseline weight, BMI, percentage of those in paid employment, and prevalence of some comorbidities as seen in Table 1. Participants who completed 12-month questionnaires were older (53.2 ± 13.9 years vs. 48.8 ± 14.0 years, *p* = 0.013), and more of them were female (76.6% vs. 62.2%, *p* = 0.018), and of Caucasian ethnicity (79.3% vs. 65.6%, *p* = 0.015), compared to those who did not have follow-up data. Similarly, the prevalence of comorbidities at baseline was higher in those who completed 12-month questionnaires, including T2DM (57.7% vs. 35.8%, *p* < 0.001), dyslipidaemia (60.4% vs. 42.4%, *p* = 0.004), and cardiovascular disease (23.4% vs. 9.3%, *p* = 0.002), with poorer glycaemic control in those who completed 12-month questionnaires (HbA1c: 7.8% vs. 6.3%, *p* = 0.001). There were no significant differences between the groups in the mean baseline EDE-QS, K10, PCS, or MCS scores as seen in Table 1.

Among the patients who completed 12 months in the program, body weight at 12 months was significantly lower (132.1 ± 26.1 kg, *p* < 0.001), when compared to baseline (140.5 ± 25.8 kg), which equated to 6.0 ± 6.8% body weight loss. The average number of clinical visits with any member of the multidisciplinary team over 12 months was 23.6 ± 9.4 visits per participant. The psychologist had reviewed 86.5% (*n* = 96) of the participants at least once, with a mean of 3.7 ± 1.9 visits per participant, while the psychiatrist had reviewed 19.8% (*n* = 22) at least once during the 12 months.

### 3.1. Risk of Eating Disorders (EDE-QS)

**Figure 2:**
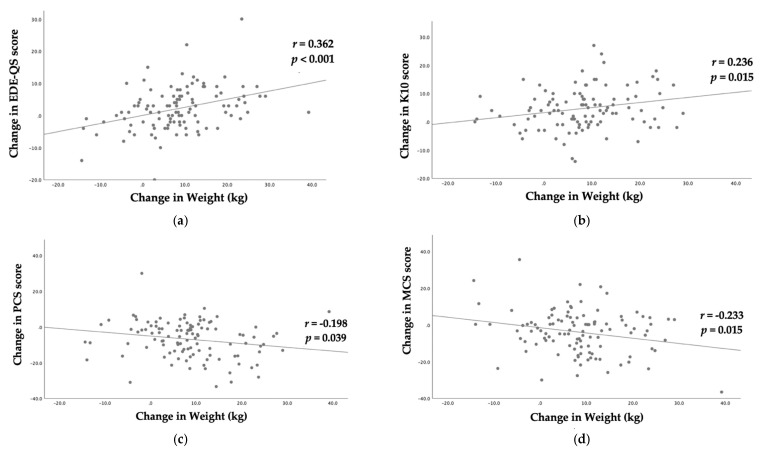
Scatter plots of change in: (**a**) Eating Disorder Examination Questionnaire Short (EDE-QS) score; (**b**) Kessler Psychological Distress Scale (K10) score; (**c**) Physical Component Summary (PCS) score; and (**d**) Mental Component Summary (MCS) score, against change in weight (kg) with fitted linear regression lines.

EDE-QS scores indicated that 53.2% (*n* = 59) of the participants had a high risk of eating disorders (cut-off score ≥ 15) at baseline, which was reduced to 46.8% (*n* = 52) at 12 months. Compared to baseline (15.7 ± 6.6), there was a statistically significant improvement in mean EDE-QS scores at 12 months (13.6 ± 6.2, *p* = 0.002) as seen in Table 2. However, the significance was borderline (*p* = 0.056) after adjusting for the change in weight using ANCOVA. The results did not change further when adjusting for number of health professional visits and weight change (*p* = 0.054). The change in EDE-QS scores from baseline to 12 months had a statistically significant positive correlation with change in weight (*r* = 0.362, *p* < 0.001) as shown in Figure 2a.

### 3.2. Psychological Distress (K10)

**Figure 3:**
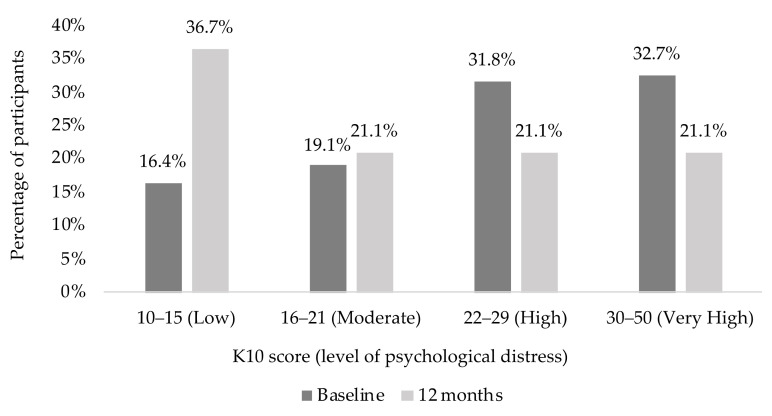
Kessler Psychological Distress Scale (K10) scores and level of psychological distress at baseline and 12 months.

Results of the K10 scores (Figure 3) suggested that 83.6% of the participants were likely to have a moderate, high or very high level of psychological distress (K10 ≥ 16) at baseline. However, this proportion dropped to 63.3% at 12 months. The K10 mean scores improved significantly at 12 months (21.2 ± 9.4, *p* < 0.001) compared to baseline (25.7 ± 9.7) as shown in Table 2. Statistically significant improvement in K10 scores was observed even when adjusted for change in weight. The results did not change when corrected for number of clinic visits in addition to correcting for weight change (*p* = 0.002). The change in K10 scores from baseline to 12 months had a statistically significant positive correlation with the change in weight (*r* = 0.236, *p* = 0.015) as shown in Figure 2b.

### 3.3. Quality of Life (SF-36)

As shown in Table 3, all eight SF-36 subscale scores of the study population at baseline and 12 months were much lower than the Australian adult population norms. The SF-36 results indicated a lower quality of life in people with class 3 obesity across all subscales. Table 3 also demonstrates that there was statistically significant improvement across all eight subscales at 12 months when compared to baseline, suggesting an improvement in HRQoL.

Compared to baseline (29.4 ± 10.1), a statistically significant improvement in PCS scores was observed at 12 months (36.1 ± 10.9, *p* < 0.001), independent of weight change and clinical visits (Table 2). Similarly, the MCS scores improved significantly at 12 months (44.0 ± 13.4, *p* = 0.001) compared to baseline (40.2 ± 12.4). The improvement was statistically significant even when adjusted for weight change (*p* = 0.040), and the results did not change when corrected for number of clinic visits in addition to correcting for weight change. The change in PCS (*r* = −0.198, *p* < 0.039) and MCS (*r* = −0.233, *p* = 0.015) scores from baseline to 12 months had a statistically significant negative correlation with the change in weight as shown in Figure 2c,d, respectively.

## 4. Discussion

This study demonstrates that a significant proportion of patients with class 3 obesity attending a weight management program are at high risk of having an eating disorder or mental health problem, and that they have a poor quality of life. A multidisciplinary approach to their management did result in the lowering of these risks and an improvement in quality of life, and this result was independent of weight loss. Given that this patient population represents those who often present for bariatric surgery or to a specialist weight management program, careful consideration of the appropriate treatment pathway for each individual is required. In addition, screening for eating disorders, psychological distress, and quality of life, as well as further support and/or psychology review, are likely to benefit such patients [50].

At presentation, 53.2% of this cohort were at risk of having an eating disorder. This is significantly higher than previous studies [19]. Lack of pre-operative assessment of eating, mood, and anxiety disorders may be associated with less impressive long-term weight loss and clinical outcomes following bariatric surgery [51]. Bariatric surgery is often preceded by a very low-calorie diet for a few weeks prior to surgery, to help shrink the liver, and is followed up by continuation of calorie restriction for a short period of time after the surgery. Hence, pre-operative screening of eating disorders in patients seeking bariatric surgery may help identify at-risk people before they start on their restricted calorie diets, and potentially result in better outcomes [52]. For example, people with a history of atypical anorexia or BED may be at risk of recurrence if they are commenced on very low-calorie diets, or undergo bariatric surgery without adequate support or preparation. Body image has also been identified as an important construct for evaluating eating psychopathology before bariatric surgery [53]. Similarly, accumulating evidence suggests the association of eating disorders with lower HRQoL [54,55]. Therefore, in order to lower the burden of obesity, research indicates the importance of a multidisciplinary approach in weight management programs, where both somatic and mental disorders are addressed [56].

Psychological distress and mental health problems have been strongly linked to obesity, with multiple studies suggesting higher rates of depression and anxiety in people with obesity, particularly class 3 obesity [57,58]. However, the high prevalence in this study of more than 83.6% being at moderate to very high risk of psychological distress at baseline is higher than previous reports [59]. While psychological distress is associated with obesity, the negative psychological, emotional, and behavioural consequences of obesity can also exacerbate psychological distress [60]. And these very high rates of psychological distress need to be taken in the context of people who may be more distressed about their weight and attending a program for help with weight loss, which may be higher than people with similar degrees of obesity that are not seeking treatment. Additionally, even though perioperative management of psychological distress while preparing for surgery may be associated with achieving better quality of life [61], there is little evidence to support delay of the surgery to achieve this.

Although obesity is linked to a lower quality of life, the scores for all aspects of quality of life in this study were much lower than the reported Australian adult population norms [49]. The individual component summary scores of the SF-36 in this cohort at baseline ranged from 23.2 to 56.1, which is much lower than the Australian adult population norms, where the scores are 64.5 to 85.1. The low SF-36 scores in this cohort were actually lower in all subscale scores than those reported for breast cancer survivors with lymphoedema where HRQoL would be expected to be much lower [62]. Previously reported SF-36 results for patients with cirrhosis and idiopathic pulmonary fibrosis have shown PCS scores comparable to our study’s cohort, while the MCS scores in our cohort were lower than those reports [63,64]. Additionally, even after a significant increase in these scores at 12 months, this cohort still had scores that were much lower than the Australian adult population norms [49]. This highlights how much the HRQoL of people with class 3 obesity is affected by their weight. Similarly, research suggests that, in addition to obesity, the presence of both eating disorders and psychological distress may further lower the HRQoL [54,55,65]. Studies have also found higher interpersonal problems in those with obesity and eating disorders which may lead to higher psychological distress, and indicate the need for inclusion of interpersonal dimensions in the evaluation of HRQoL [66,67,68]. Our study found that improvements in eating disorder risk, psychological distress, and HRQoL were achievable through a multidisciplinary weight management program. These findings support the need for a focus on improving HRQoL, as well as routine screening for eating disorders and mental health problems in people with class 3 obesity, including those who present for bariatric surgery, as these measures may help to improve post-operative outcomes [69].

Weight loss over 12 months in this high-risk population was shown to be modest (6%) and was comparable to previously published studies on non-surgical weight loss [39,70]. Although the link between obesity and eating disorders, psychological distress, and poor quality of life is well documented [59,71,72], what is less clear is the effect of modest non-surgical weight loss on these conditions. The improvement in K10, PCS, and MCS scores at 12 months remained significant, even after correcting for weight change. This suggests an additional benefit beyond weight loss when attending a multidisciplinary program, in that psychological distress and quality of life can be improved, which may be partly due to patients receiving unbiased support for their weight loss attempts, and thus patients not feeling judged. Obesity stigma and negative attitudes, and weight bias against people with obesity have been well documented among general practitioners, medical professionals, and nursing staff [73], with bullying victimization often reported by people with severe obesity [74]. Therefore, the safe environment of the program in this study may have helped the patients beyond just weight loss. Further, patients will likely have derived additional mental health benefits from the peer support and vicarious learning they experienced while attending the program. People with class 3 obesity often have complex physical as well as mental health comorbidities, and management is often complex and beyond simple advice about activity and food intake [75]. This study supports the notion that weight loss alone should not be a marker of success when it comes to the management of class 3 obesity, as is often the case. Moreover, assessment of weight alone distracts from observing other health benefits of a multidisciplinary weight management program.

The main limitation of this study was that follow-up data were not available in a large proportion of the patients, some of which were due for collection during COVID-19 restrictions. However, there were no major differences in the questionnaire scores or BMI at baseline when comparing those who completed questionnaires at 12 months with those who did not or were lost to follow-up. Moreover, although some of the 12-month scores were collected in the challenging times of COVID-19 restrictions, there was still an improvement in all scores, despite the potential for lower scores at the 12-month follow-up. Another limitation was that this study only used questionnaires to assess the risk of eating disorders, psychological distress, or quality of life, rather than interviews to assess risk or make a clinical diagnosis. Further work with control groups to compare outcomes and better follow-up would be desirable. Future research could further explore the risk of eating disorders, level of psychological distress, and HRQoL in people with class 3 obesity using interviews by health professionals rather than self-reporting. Furthermore, future research could explore body image as a construct and interpersonal dimension in HRQoL. We used validated questionnaires but, as with any questionnaire, they may have been subject to recall bias or reporting bias. However, the current study was conducted in a real-life clinical program and, unlike most clinical trials, did not have extensive inclusion/exclusion criteria. All patients enrolled in the weight management program who had class 3 obesity were included in this study and were aged ≥18 years with BMI ≥ 40 kg/m^2^. Therefore, it would be possible for this study to be replicated in other real-life clinical settings managing people with class 3 obesity.

## 5. Conclusions

This study demonstrates that a significant proportion of patients with class 3 obesity attending a weight management program are at high risk of having an eating disorder or mental health problem, and that they have a very poor HRQoL. The EDE-QS score improved significantly at 12 months but became borderline significant when corrected for weight change. The improvement in K10, PCS, and MCS scores at 12 months remained significant, after correcting for weight change. The findings of this study suggest that, in the management of people with class 3 obesity, rather than focusing solely on weight loss, it is important to adopt a multidisciplinary approach. This may help to reduce the risk of eating disorders and psychological distress, and improve HRQoL. Given the high prevalence shown in this study, people being considered for bariatric surgery or attending a specialist weight management program should also be routinely screened for eating disorder risk, psychological distress, and health-related quality of life.

## Figures and Tables

**Table 1:** Baseline characteristics of the study participants.

Variable (Mean ± SD or %)	All Participants (*n* = 262)	Participants with 12-Month Follow-Up and Questionnaire Data (*n* = 111)	Participants with no 12-Month Follow-Up or Questionnaire Data (*n* = 151)	*p*-Value
Age (years)	50.7 ± 14.0	53.2 ± 13.9	48.8 ± 14.0	0.013 *
Female	180 (68.7%)	85 (76.6%)	95 (62.2%)	0.018 *
Caucasian ethnicity	187 (71.4%)	88 (79.3%)	99 (65.6%)	0.015 *
Weight (kg)	143.0 ± 30.0	140.5 ± 25.8	144.9 ± 32.9	0.256
BMI (kg/m^2^)	51.2 ± 9.0	51.27 ± 8.57	51.1 ± 9.3	0.930
In paid employment	77 (29.4%)	32 (28.8%)	45 (29.8%)	0.864
Hypertension	168 (64.1%)	77 (69.4%)	91(60.3%)	0.129
Type 2 diabetes mellitus	118 (45.1%)	64 (57.7%)	54 (35.8%)	<0.001 *
Dyslipidaemia	131 (50.0%)	67 (60.4%)	64 (42.4%)	0.004 *
Cardiovascular disease	40 (15.3%)	26 (23.4%)	14 (9.3%)	0.002 *
Fatty liver disease	59 (22.5%)	21 (18.9%)	38 (25.2%)	0.232
Obstructive sleep apnoea	135 (51.5%)	60 (54.0%)	76 (50.3%)	0.551
Gastroesophageal reflux disease	114 (43.5%)	44 (39.6%)	70 (46.4%)	0.278
Thyroid disorder	52 (19.9%)	26 (23.4%)	26 (17.2%)	0.213
Cholesterol (mmol/L)	4.6 ± 2.9	4.5 ± 1.1	4.6 ± 3.8	0.753
Triglyceride (mmol/L)	1.9 ± 1.1	1.9 ± 1.0	2.0 ± 1.3	0.602
HDL (mmol/L)	1.2 ± 0.3	1.2 ± 0.3	1.1 ± 0.3	0.052
LDL (mmol/L)	2.2 ± 0.9	2.5 ± 1.1	2.3 ± 0.8	0.315
ALT (IU/L)	31.7 ± 18.7	27.0 ± 14.8	33.3 ± 21.2	0.130
AST (IU/L)	26.5 ± 12.1	26.5 ± 11.6	26.5 ± 12.5	0.972
GGT (IU/L)	48.4 ± 46.2	46.4 ± 41.9	50.0 ± 49.4	0.554
HbA1c (%)	7.0 ± 5.4	7.8 ± 7.8	6.3 ± 1.4	0.001 *
EDE-QS score	16.2 ± 6.7	15.7 ± 6.6	16.6 ± 6.8	0.286
K10 score	26.0 ± 9.6	25.7 ± 9.7	26.2 ± 9.6	0.711
PCS score	30.8 ± 10.3	29.4 ± 10.1	31.7 ± 10.4	0.075
MCS score	39.3 ± 11.8	40.2 ± 12.4	38.7 ± 11.3	0.319

* Significant at *p* < 0.05. BMI: Body mass index. HDL: High-density lipoprotein. LDL: Low-density lipoprotein. ALT: Alanine aminotransferase. AST: Aspartate aminotransferase. GGT: Gamma-glutamyl transferase. HbA1c: Glycated haemoglobin A1c. EDE-QS: Eating Disorder Examination Questionnaire Short. K10: Kessler Psychological Distress Scale. PCS: Physical Component Summary. MCS: Mental Component Summary.

**Table 2:** Mean EDE-QS, K10, PCS, and MCS scores at baseline and 12 months.

Score (Mean ± SD)	Baseline	12 Months	Unadjusted *p*-Value	*p*-Value Adjusted for Change in Weight
EDE-QS	15.7 ± 6.6	13.6 ± 6.2	0.002	0.056
K10	25.7 ± 9.7	21.2 ± 9.4	<0.001	0.002
PCS	29.4 ± 10.1	36.1 ± 10.9	<0.001	<0.001
MCS	40.2 ± 12.4	44.0 ± 13.4	0.001	0.040

EDE-QS: Eating Disorder Examination Questionnaire Short. K10: Kessler Psychological Distress Scale. PCS: Physical Component Summary. MCS: Mental Component Summary.

**Table 3:** SF-36 subscale scores of study participants at baseline and 12 months, with *p*-values, compared with the Australian adult population norms.

36-Item Short Form Survey (SF-36) Subscales	Australian Adult Population Norms (Mean) [49]	Study Participants (Mean ± SD)		*p*-Value
		Baseline	12 Months	
Physical Function (PF)	83.5	32.3 ± 25.2	50.2 ± 29.2	<0.001
Role limit Physical (RP)	80.3	23.2 ± 33.1	45.4 ± 43.2	<0.001
Role limit Emotional (RE)	83.2	40.5 ± 41.5	54.0 ± 43.4	0.003
Vitality (VT)	64.5	31.3 ± 18.8	44.6 ± 23.2	<0.001
Mental Health (MH)	76.0	56.1 ± 23.6	62.6 ± 23.8	0.003
Social Function (SF)	85.1	44.6 ± 27.8	61.7 ± 31.1	<0.001
Bodily Pain (BP)	76.9	38.6 ± 23.8	45.9 ± 27.2	<0.001
General Health (GH)	71.8	29.6 ± 18.1	44.6 ± 21.2	<0.001

## Data Availability

The data used to support the findings of this study are available from the corresponding author upon request.
